# Antiangiogenic and tumour inhibitory effects of downregulating tumour endothelial FABP4

**DOI:** 10.1038/onc.2016.256

**Published:** 2016-08-29

**Authors:** U Harjes, E Bridges, K M Gharpure, I Roxanis, H Sheldon, F Miranda, L S Mangala, S Pradeep, G Lopez-Berestein, A Ahmed, B Fielding, A K Sood, A L Harris

**Affiliations:** 1 Hypoxia and Growth Factor Group, WIMM, Department of Oncology, University of Oxford, Oxford, UK; 2 Department of Gynecologic Oncology, University of Texas, Austin, TX, USA; 3 Department of Cellular Pathology, Oxford University Hospitals and NIHR Biomedical Research Centre Oxford, John Radcliffe Hospital, Oxford, UK; 4 Ovarian Cancer Cell Laboratory, WIMM, Nuffield Department of Obstetrics and Gynaecology, University of Oxford, Oxford, UK; 5 Center for RNA Interference and Non-Coding RNA, MD Anderson Cancer Center, Department of Gynecologic Oncology, University of Texas, Austin, TX, USA; 6 Department of Cancer Biology, University of Texas, Austin, TX, USA; 7 Department of Nutritional Sciences, University of Surrey, Surrey, UK

## Abstract

Fatty acid binding protein 4 (FABP4) is a fatty acid chaperone, which is induced during adipocyte differentiation. Previously we have shown that FABP4 in endothelial cells is induced by the NOTCH1 signalling pathway, the latter of which is involved in mechanisms of resistance to antiangiogenic tumour therapy. Here, we investigated the role of FABP4 in endothelial fatty acid metabolism and tumour angiogenesis. We analysed the effect of transient FABP4 knockdown in human umbilical vein endothelial cells on fatty acid metabolism, viability and angiogenesis. Through therapeutic delivery of siRNA targeting mouse FABP4, we investigated the effect of endothelial FABP4 knockdown on tumour growth and blood vessel formation. *In vitro*, siRNA-mediated FABP4 knockdown in endothelial cells led to a marked increase of endothelial fatty acid oxidation, an increase of reactive oxygen species and decreased angiogenesis. *In vivo*, we found that increased NOTCH1 signalling in tumour xenografts led to increased expression of endothelial FABP4 that decreased when NOTCH1 and VEGFA inhibitors were used in combination. Angiogenesis, growth and metastasis in ovarian tumour xenografts were markedly inhibited by therapeutic siRNA delivery targeting mouse endothelial FABP4. Therapeutic targeting of endothelial FABP4 by siRNA *in vivo* has antiangiogenic and antitumour effects with minimal toxicity and should be investigated further.

## Introduction

The metabolism of fatty acids (FA) has been recognized as a key process required for the formation of new blood vessels (angiogenesis).^1^ FAs can be used for membrane and lipid synthesis, regulation of gene expression and energy production.^2, 3, 4, 5^ Via the TCA cycle, the oxidation of FAs (FAO) significantly contributes to the *de novo* synthesis of nucleotides during endothelial cell (EC) proliferation.^1^ Upon uptake and release of FAs into the cell, FAs are either activated by acyl-CoA synthases or bound by FA-binding proteins 1–9 (FABP1–9). However, FAs, which are not converted to FA-CoA or bound to FABPs, can cause cellular stress.^6^ Adipocyte FABP4 binds FAs and provides feedback inhibition of lipolysis through interaction with lipases. FABP4−/− adipocytes, in which this feedback inhibition is lost, show higher lipolytic rates and higher levels of intracellular free FAs.^7, 8^ In addition, FABP4 delivers FAs to the nucleus to activate peroxisome proliferator-activated nuclear receptor γ, a main transcriptional inducer of adipocyte FA storage. FA metabolism is deregulated in a number of cancers.^5, 9^ Specifically, FABPs are involved in tumour biology through their function in regulation of PPAR activity and/or FA uptake and oxidation.^3, 4, 10^ Interestingly, in FABP4−/− mice, ovarian tumour xenograft growth and metastasis were reduced.^11^ This was linked to reduced utilization of adipocyte-derived FAs in tumour cells and to reduced FABP4 expression in adipocytes and adjacent tumour cells.^11^

Vascular endothelial growth factor A (VEGFA) is a major inducer of tumour blood vessel growth (tumour angiogenesis). Indeed, vessel density in ovarian tumours increases with tumour progression and correlates with expression of VEGF receptor 2.^12^ However, many ovarian tumours are not responsive to anti-VEGFA therapies due to upregulation of alternative pathways. As such, NOTCH1 signalling induction by its ligand Delta-like ligand 4 (DLL4), itself a target of VEGA, is associated with anti-VEGFA resistance in ovarian cancer patients.^13^ NOTCH1 signalling limits angiogenesis by modulating the VEGFA response, leading to larger, better perfused tumour vessels, less hypoxia and reduced response to VEGFA-targeting therapies.^14^ Target genes of NOTCH1 and VEGFA signalling, such as the NOTCH1 targets Jagged 1 and HESR1 and the VEGFA target matrix metallopeptidase 9, are upregulated in tumour-associated ECs from invasive ovarian carcinoma.^15^ Dual targeting of DLL4/NOTCH1 and VEGFA signalling is more effective than single therapy in orthotopic mouse ovarian tumour models,^16^ making NOTCH1 and VEGFA signalling an attractive target in ovarian tumour angiogenesis.

We have shown that in ECs, VEGFA upregulates FABP4 expression indirectly by inducing DLL4, which activates NOTCH1 signalling and initiates *FABP4* gene transcription.^17^ However, tumour angiogenesis in FABP4−/− mice, and its relation to NOTCH1/ VEGFA signalling pathways and endothelial FA metabolism, has not been studied in detail.^11^ In this study, we aimed to investigate the role of FABP4 in ovarian tumour angiogenesis and in EC FA metabolism. We show that endothelial FABP4 expression requires NOTCH1 and VEGFA signalling, and is required for ovarian tumour angiogenesis. Furthermore, endothelial FABP4 silencing led to deregulation of enzymes governing FA storage and lipolysis, and increased the rate of FAO. FAO is required for vascular sprouting and contributes to NADPH and reactive oxygen species (ROS) scavenging in certain cell types.^1, 18^ However, we found that FABP4 silencing, while increasing FAO, led to decreased sprouting. FABP4 silenced cells showed an increased mitochondrial membrane potential, which was dependent on increased FAO. We conclude that FABP4 is required for the regulation of free FA levels in the cell to protect from FA-induced ROS production in ECs, and that FABP4, a target of VEGF and NOTCH signalling, plays a significant role in the formation of tumour vasculature, controlling ROS formation and intracellular FA trafficking in FA-rich environments.

## Results

### Activation of endothelial NOTCH1 signalling *in vivo* induces FABP4 expression

**Figure 1:**
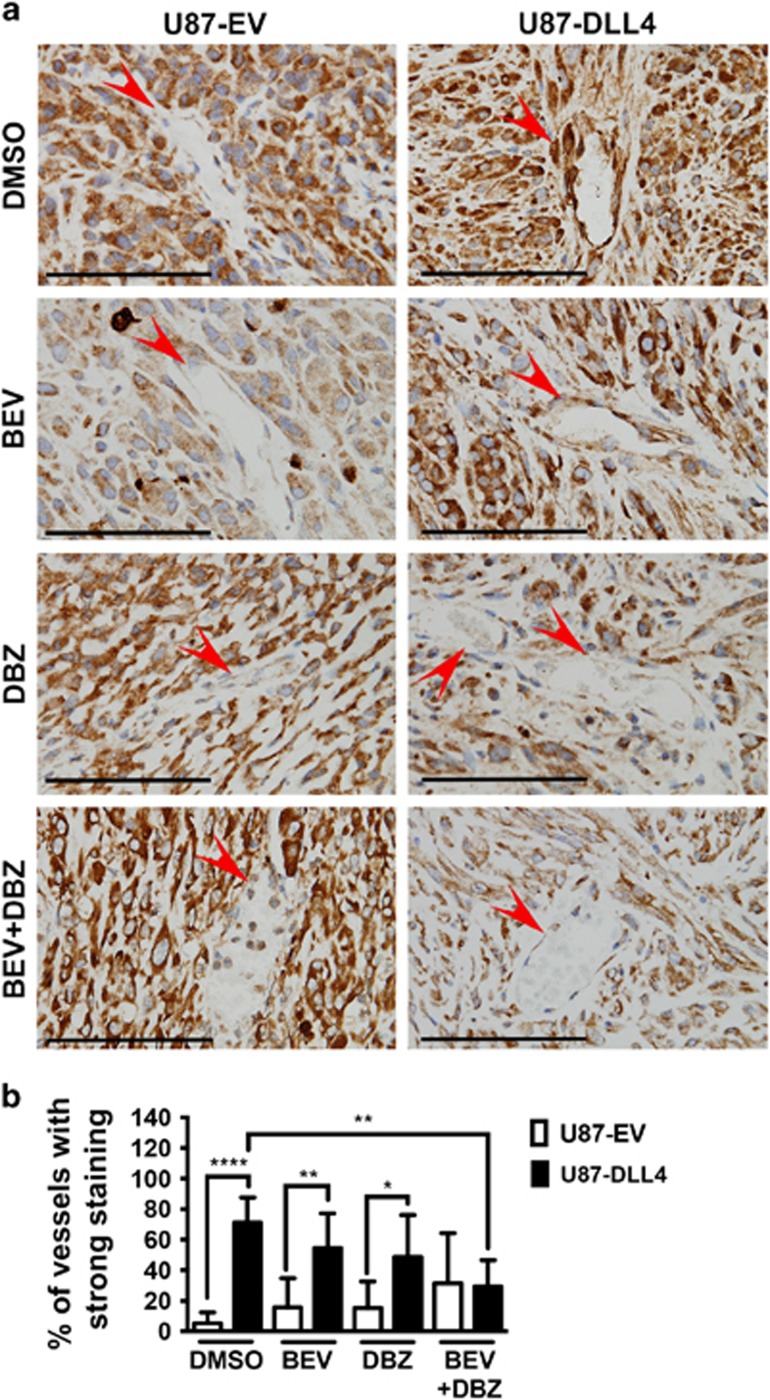
DLL4-overexpressing glioblastoma xenografts show increased vascular FABP4 expression that is sensitive to drug inhibition of NOTCH1 and VEGFA signalling. Formaldehyde fixed-paraffin embedded sections of empty vector (EV) control or DLL4-overexpressing U87 xenografts treated with vehicle control (dimethylsulphoxide, DMSO), the VEGFA-antibody bevacizumab (BEV) and/or the γ-secretase inhibitor dibenzazepine (DBZ, inhibits NOTCH1 signalling) were stained for FABP4 using a mouse and human specific antibody (**a**), and vascular FABP4 staining (red arrow) was quantified. The percentage of strongly positive vessels is shown (**b**). Scale bar=100 μm, *n*=5 per group, *P<0.05; **P<0.01; ****P<0.00001. Error bars, s.d.

We have previously shown that vessels in DLL4-overexpressing U87 xenografts are larger, better perfused and the tumours less hypoxic, due to increased NOTCH1 signalling in the vasculature.^14^ These xenografts were chosen to study tumour endothelial FABP4 expression in response to NOTCH1 signalling and NOTCH1/ VEGFA-targeting therapies *in vivo*. In these xenografts, we found that more than 50% of the vessels in DLL4-overexpressing xenografts showed a strong FABP4 expression compared with 10–20% of the vessels in the control (empty vector, EV) tumours (*P<*0.00001) (Figures 1a and b). In tumours treated with bevazicumab (BEV, antibody targeting VEGFA) or dibenzazepine (DBZ, inhibits NOTCH1 signalling by inhibiting γ-secretase), FABP4 staining in the vessels of DLL4-overexpressing tumours was still stronger and more extensive than in the vessels of EV tumours (*P<*0.01 in BEV-treated, *P<*0.05 in DBZ-treated tumours). In tumours treated with BEV and DBZ combined, vessels with strong FABP4 staining were significantly less than in dimethylsulphoxide-treated DLL4-overexpressing tumours, and no difference to EV tumours was observed (Figures 1a and b). This shows that tumour endothelial FABP4 expression is induced by activation of NOTCH1 signalling, and can be targeted by inhibition of VEGFA and NOTCH1 signalling.

### Vascular FABP4 expression is associated with low-grade ovarian cancer

**Figure 2:**
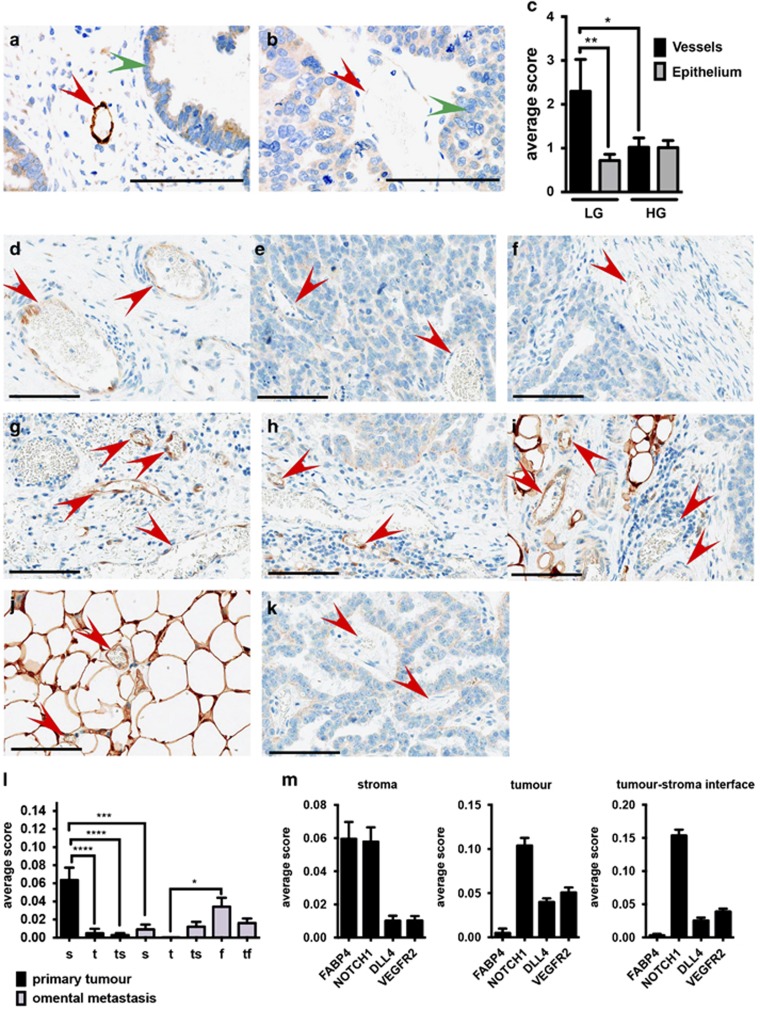
Vessels of low-grade serous ovarian carcinoma show overall strongly positive FABP4 expression. Tissue microarrays of low- and high-grade ovarian serous carcinoma immunohistochemistry were performed for FABP4 using a mouse and human specific antibody ((**a**) low-grade and (**b**) high-grade; vessels are indicated with a red arrow, epithelial cells with a green arrow), and vascular FABP4 staining was quantified. The average combined score of intensity and extent of vascular versus epithelial FABP4 expression is shown (**c**). Paired patient samples of primary and metastatic serous ovarian carcinoma were selected and immunohistochemistry was performed for FABP4. Intensity and extent of vascular FABP4 expression (red arrow) were quantified in vessels of the stroma (s) ((**d**) primary tumour and (**g**) metastatic tumour), within the tumour epithelium (t) ((**e**) primary tumour and (**k**) metastatic tumour), within tumour–stroma interface (ts) ((**f**) primary tumour and (**h**) metastatic tumour), the fat tissue (f) (**j**), the tumour–fat interface (tf) (**i**) of the primary and metastatic tumour. The average combined score of intensity and extent of vascular FABP4 expression in these different compartments is shown (**l**) and compared with NOTCH1, DLL4 and VEGFR2 expression in compartments of the primary tumour (**m**). Scale bar=100 μm, *n*=7 paired samples, *P<0.05; **P<0.01; ***P<0.0001, ****P<0.00001. Error bars, s.d.

Patients with advanced serous ovarian carcinoma show increased expression of FABP4 in tumour cells metastasized to the omentum compared with cells of the primary tumour.^11^ NOTCH1 and VEGFA signalling are implicated in the progression of ovarian tumour angiogenesis.^12, 13, 15, 16^ However, studies investigating the expression in tumour vessels are lacking. We obtained tumour microarrays of 16 low-grade and 51 high-grade serous ovarian carcinomas and determined expression of FABP4 protein in tumour vessels and tumour epithelial cells. Epithelial FABP4 expression in low-grade and high-grade carcinomas was similar in intensity and extent. However, vascular FABP4 expression in low-grade carcinomas was more than two-fold higher than FABP4 in the vessels of high-grade tumours (Figures 2a–c).

### Vascular FABP4 expression is highest in the stromal compartment of ovarian tumours

We then studied the expression of FABP4 in vessels of high-grade primary ovarian tumours and omental metastasis in more detail, focusing on the intratumoural distribution of FABP4-positive vessels. We observed that in the primary tumour, FABP4 expression in vessels within the stroma (Figures 2d and l) was significantly higher than in vessels directly adjacent to tumour cells (Figures 2e and l) and vessels in the tumour–stroma interface (Figures 2f and l). Vascular FABP4 expression in the stroma of the primary tumour was also significantly higher than in the stroma of omental metastasis (Figure 2g). In omental metastasis, we observed similar FABP4 expression in the vessels of the stroma (Figure 2g), the tumour–stroma interface (Figure 2h) and the tumour–fat interface (Figure 2i). The expression of FABP4 in vessels of the fat tissue distant from the tumour (Figure 2j) was significantly higher than in vessels directly adjacent to tumour cells (Figure 2k).

These data show that FABP4 expression was highest in stroma- and fat tissue-embedded vessels, in which proangiogenic signals coming from the tumour might be less prominent. Indeed, in the stromal regions of the primary tumour, the vascular expression of cleaved NOTCH1 (NOTCH1 intracellular domain, NICD, indicates active NOTCH1 signalling) correlated with FABP4 expression, whereas in vessels within the tumour and the tumour–stroma interface this correlation was lost. Instead, components of the VEGF signalling pathway were more prominent, with DLL4 and VEGFR2 being more highly expressed (Figure 2m, representative images shown in Supplementary Figure S1).

### Mouse FABP4 knockdown decreases ovarian tumour xenograft growth and microvessel density

FABP4 expression was highest in vessels of low-grade serous carcinoma, and in the stromal compartment of advanced serous carcinoma, challenging the question whether tumour endothelial FABP4 expression is required for tumour progression. Previous studies have not investigated the effect of endothelial FABP4 knockdown (KD) on tumour angiogenesis and tumour growth.^11^ We utilized the chitosan-nanoparticle drug delivery system, which has a high potential for clinical application, to deliver two different small interfering RNA (siRNA) sequences targeting mouse *FABP4* (Supplementary Figure S3) in an orthotopic model of ovarian carcinoma.^19, 20^ This system efficiently targets blood vessels without affecting other stromal cells such as infiltrating macrophages.^19^ By using the chitosan-nanoparticle system, we were able to achieve efficient silencing of FABP4 in the tumour vessels while not affecting FABP4 expression in stromal or adipose cells (Supplementary Figure S3).

**Figure 3:**
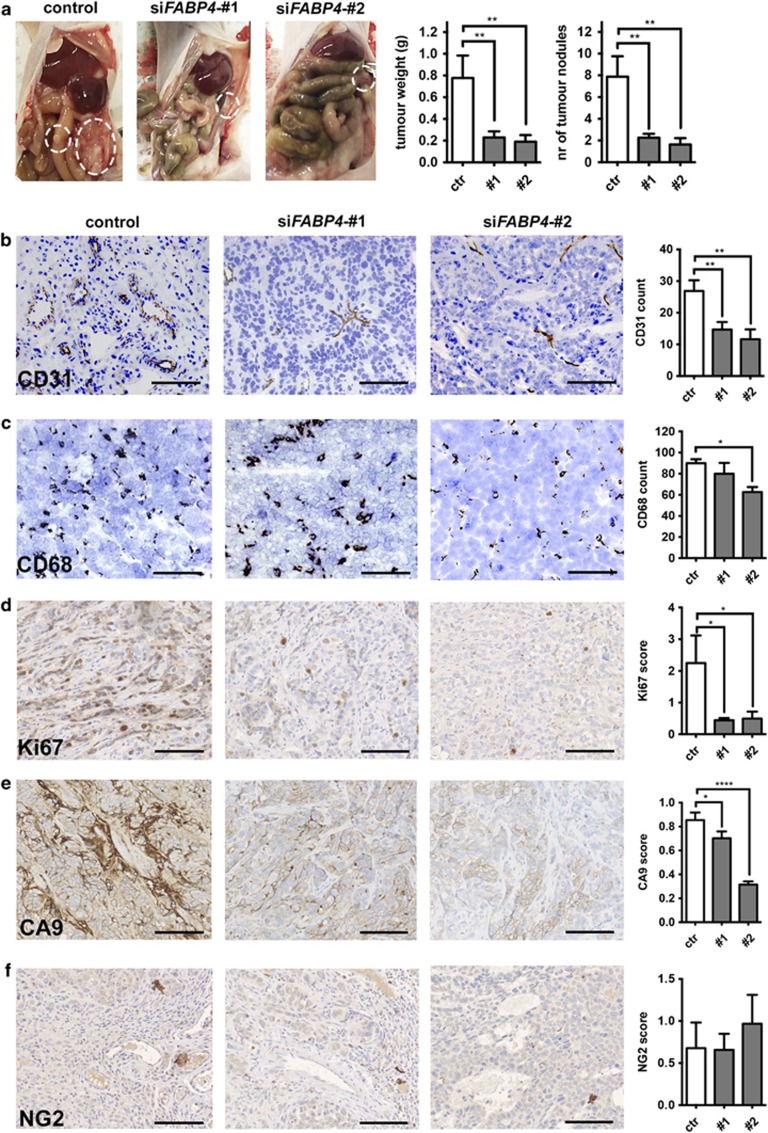
Therapeutic siRNA-mediated knockdown of FABP4 in the mouse stroma decreases tumour growth and tumour angiogenesis. Chitosan-nanoparticle delivery of two siRNA sequences targeting mouse *FABP4* was carried out in mice bearing SKOV3 ip2 orthotopic xenografts. Number of tumour nodules and tumour weight were determined (**a**), and immunohistochemistry for CD31 (**b**), CD68 (**c**), Ki67 (**d**), CA9 (**e**) and NG2 (**f**) was performed on tumour formaldehyde fixed-paraffin embedded sections and quantified. Scale bar=100 μm. *n*=10 per group, *P<0.05; **P<0.01; ****P<0.00001. Error bars, s.e.m.

We observed a significant inhibition of tumour growth with mouse FABP4 KD; a <70% reduction of tumour weight and number of tumour nodules with both siRNA treatments (Figure 3a). Strikingly, microvessel density was reduced by >50% with both treatments compared with the control (Figure 3b). No consistent effects on macrophage infiltration were detected (Figure 3c). Ki67 as a marker for proliferation was significantly downregulated in tumour cells grown in xenografts treated with si*FABP4* (Figure 3d). There was significantly reduced hypoxia in si*FABP4*-treated tumours (Figure 3e). This was accompanied by a reduction of necrotic areas (Supplementary Figure S4), which is likely a result of the decrease in tumour size and support of the viable rim by existing host blood vessels. The expression of the pericyte marker neural/glial antigen 2 (NG2) showed no significant change in the treated xenografts (Figure 3f).

### FABP4 silencing reduces endothelial sprout elongation *in vitro*

**Figure 4:**
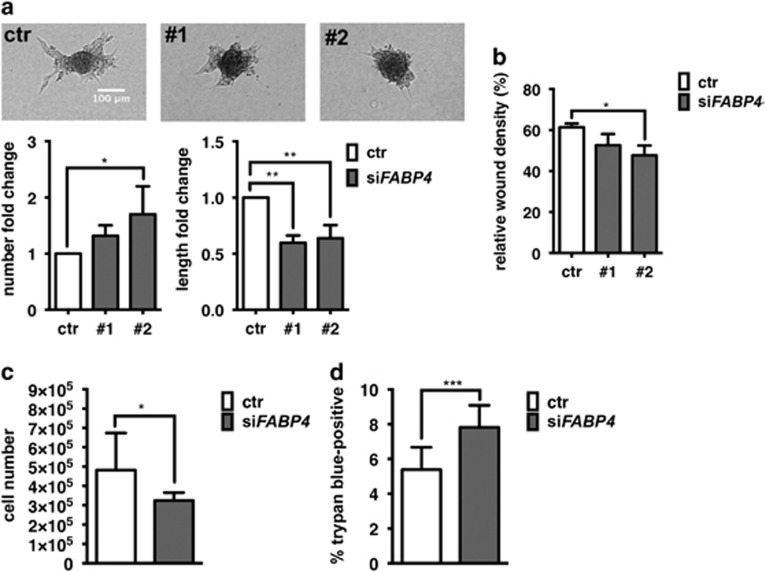
FABP4 knockdown reduces vascular sprout elongation and endothelial cell viability. Human umbilical vein endothelial cells (EC) were transfected with non-targeting control siRNA or two different sequences of siRNA targeting FABP4 (20 nm, pooled or single as indicated). To determine vascular sprouting, transfected or ECs were grown as spheroids in hanging drops and embedded in matrigel, and sprout number and length were quantified after 48 h. *n*=4 (**a**). To determine migration, scratched confluent monolayers were monitored for % wound closure over time (**b**). To measure cell number as an indicator for proliferation, ECs transfected with pooled siRNA sequences #1 and #2 targeting FABP4 were grown for 72 h, trypsinized and counted. *n*=3 (**c**). Cell viability was determined in the same samples based on cell membrane permeability for trypan blue. *n*=4 (**d**). *P<0.05; **P<0.01; ***P<0.001. Error bars, s.d.

The interplay of VEGF and DLL4–NOTCH1 signalling pathways controls vascular sprouting, that is, DLL4–NOTCH1 limits the VEGF-induced migratory tip cell phenotype and enables the elongation of the sprout via induction of the more proliferative stalk cell phenotype. Inhibition of VEGF signalling abrogates sprout formation, whereas inhibition of NTOCH1 signalling leads to enhanced tip cell formation but reduced sprout elongation.^21^ FABP4, being a target of both VEGF and NOTCH, may be required for vascular sprouting. Indeed, FABP4 silencing by siRNA in human umbilical vein endothelial cells (EC) led to a reduction of vascular sprouting. While sprout number was overall not affected or slightly increased, we observed a significant reduction of sprout length by 50% (Figure 4a). EC migration was overall not consistently changed in FABP4 silenced ECs (Figure 4b), contrary to previous reports.^22^ EC proliferation, required for elongation of the sprout, was significantly decreased in FABP4 silenced ECs (Figure 4c). Since this was accompanied by increased cell death (Figure 4d), the sprouting defect is likely due to a decreased capacity of FABP4 silenced ECs to elongate the sprout.

### FABP4 silencing increases endothelial FAO *in vitro*

**Figure 5:**
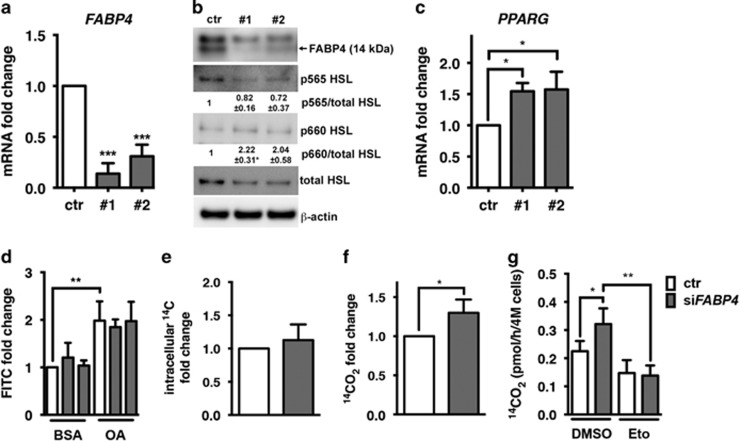
FABP4 knockdown regulates fatty acid metabolism enzymes and increases fatty acid oxidation. Human umbilical vein endothelial cells (EC) were transfected with non-targeting control siRNA or two different sequences of siRNA targeting FABP4 (20 nm) and mRNA expression of *FABP4* and *PPARG* were determined and are expressed relative to β-actin (ACTB). *n*=5 (**a, b**). Protein levels of FABP4, phosphorylated HSL (Serine 565, p565, inhibitory; Serine 660, p660, activating) and total HSL were analysed in response to FABP4 knockdown by immunoblot analysis, using β-actin as a loading control. Band densitometry analysis of phosphorylated HSL was carried out relative to total HSL. *n*=3 (**c**). To measure lipid droplet accumulation, transfected ECs were exposed to media containing BSA or oleic acid (OA, serving as a positive control) conjugated to BSA for 16 h prior to staining lipid droplets with the fluorescent dye LD540 and measuring signal intensity in the fluorescein isothyanate (FITC) channel by FACS analysis. *n*=3 (**d**). Fatty acid uptake and oxidation were measured by detecting intracellular ^14^C or ^14^CO_2_ derived from [U14C]-labelled OA in the cell culture media. *n*=4 (**e–g**). FAO rates were corrected for the levels of FAs taken up into the cells. Etomoxir (50 μm, Eto) or dimethylsulphoxide (DMSO) were added 16 h after the transfection to inhibit fatty acid oxidation *n*=3 (**g**) *P<0.05; **P<0.01; ***P<0.001. Error bars, s.d.

FABP4 has been implicated in the transport and release of FAs from lipid stores, by providing feedback inhibition to hormone-sensitive lipase (HSL) when intracellular free FAs are abundant, and by downregulating *PPARG* mRNA expression.^7, 23^ When FABP4 was silenced in ECs (Figures 5a and b) we detected relatively decreased levels of serine 565 phosphorylation, inhibiting HSL activity (Figure 5b), and relatively increased levels of serine 660 phosphorylation, stimulating HSL activity (Figure 5b), indicating overall increased activity of HSL (Figure 5b).

An increase of lipolysis is likely to result in depletion of intracellular lipid stores. Vice versa, increased lipolysis may lead to a toxic increase of intracellular free FAs and upregulation of rescue mechanisms, such as activation of lipid synthesis and storage. Channelling of FAs into lipid stores can prevent toxic levels of intracellular free FAs. Indeed, we detected upregulation of *PPARG* mRNA expression, pointing to an activation of FA storage (Figure 5c). However, the levels of lipid stores remained unchanged in FABP4 silenced ECs (Figure 5d). Furthermore, we investigated the uptake and accumulation of FAs in ECs by measuring intracellular accumulation of exogenous [U^14^C]-oleic acid. However, FABP4 silencing had no effect on the accumulation of intracellular [U^14^C]-oleic acid (Figure 5e). Excess intracellular FAs can also be eliminated by degradation through FAO. To test this, we measured the rate of oxidation of [U^14^C]-labelled oleic acid in FABP4 silenced conditions. Indeed, FABP4 silencing led to an increase of FAO by 40%, which was reversed by etomoxir, an inhibitor of FA import into the mitochondria (Figures 5f and g).

### FABP4 silencing causes increased ROS production

**Figure 6:**
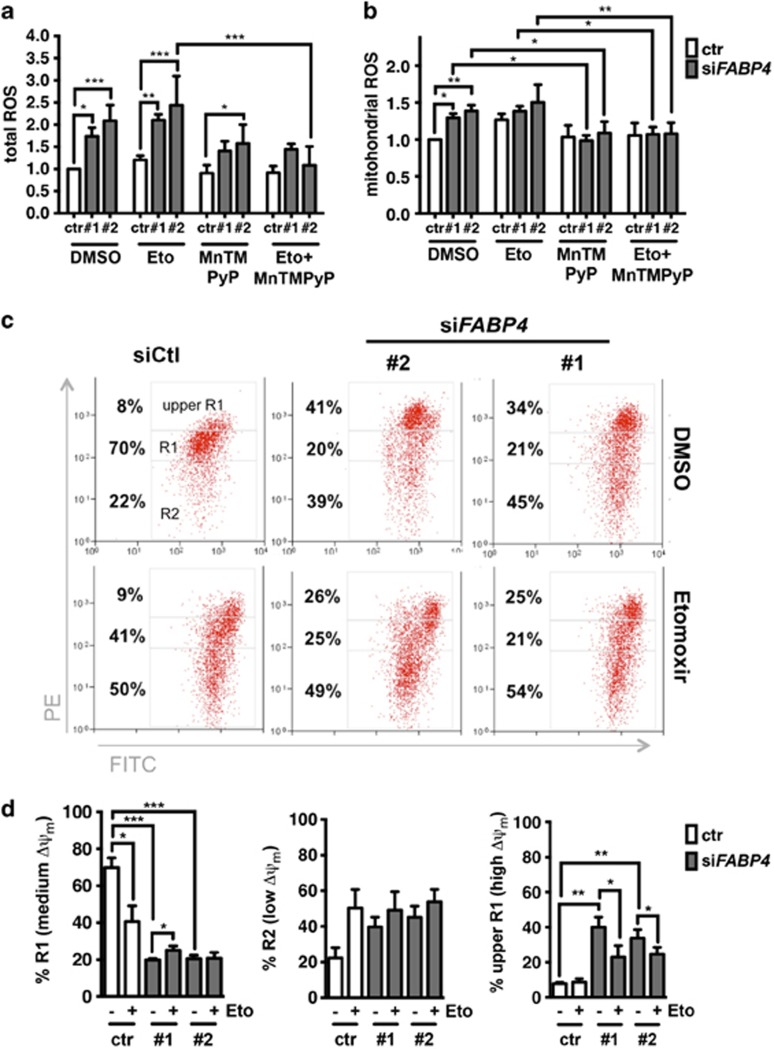
FABP4 knockdown leads to upregulation of reactive oxygen species (ROS) and mitochondrial membrane potential (Δ*Ψ*_m_). For ROS measurements, transfected samples were treated with etomoxir (Eto, inhibits mitochondrial fatty acid import) and/or the superoxide dismutase mimetic manganese (III) tetrakis(1-methyl-4-pyridyl)porphyrin pentachloride (MnTMPyP, 12.5 μm) or dimethylsulphoxide (DMSO) as a vehicle control for 72 h. Total ROS were measured by using 5-(and-6)-chloromethyl-2′,7′-dichlorodihydrofluorescein diacetate (CM-H2DCFDA; Life Technologies). *n*=3 (**a**). Mitochondrial ROS were measured by FACS using MitoSOX Red mitochondrial superoxide indicator (Life Technologies). *n*=3 (**b**). Representative images of FACS analysis and gating strategy of JC1 staining to determine Δ*Ψ*_m_ are shown. JC1 is a cationic dye that forms aggregates, exhibiting a green-to-red shift when the Δ*Ψ*_m_ is increased. The upper R1 quadrant represents high Δ*Ψ*_m_, R1 represents medium Δ*Ψ*_m_ and R2 represents low Δ*Ψ*_m_ (**c**). Quantification of biological replicates is shown. *n*=5 (**d**). *P<0.05; **P<0.01; ***P<0.001. Error bars, s.d.

ECs with FABP4 KD exhibited signs of cellular damage. Since increased mitochondrial respiration and abnormally increased FAO can lead to an increase of mitochondrial ROS,^24^ causing cellular toxicity, we quantified total and mitochondrial ROS levels in FABP4 KD ECs. We treated ECs with the superoxide dismutase mimetic MnTMPyP to scavenge mitochondrial ROS and/or etomoxir to inhibit FAO. Indeed, FABP4 silencing led to a two-fold increase of total ROS, which was partly reversed by MnTMPyP. Total ROS were not rescued by etomoxir treatment (Figure 6a). Mitochondrial ROS were significantly increased by 20–30% in FABP4 KD ECs, which was completely reversed by MnTMPyp. The increase in mitochondrial ROS in FABP4 silenced conditions was not observed when FAO was inhibited (Figure 6b).

Mitochondrial ROS production can lead to decreased mitochondrial function and cytosolic oxidation, contributing to cellular damage. Mitochondrial ROS are produced from the leakage of electrons passing through the electron transport chain during mitochondrial respiration, which leads to partial reduction of oxygen to produce superoxide at complex I and II, and subsequent superoxide dismutation to H_2_O_2_ by superoxide dismutase. While superoxide can hardly pass the outer mitochondrial membrane, H_2_O_2_ can be transported into the cytosol and lead to oxidative modifications. Electron transport chain activity is coupled to the pumping of protons, leading to the formation of the mitochondrial membrane potential (Δ*Ψ*_m_). At higher Δ*Ψ*_m_ ROS production increases exponentially.^25^ We measured Δ*Ψ*_m_ to determine mitochondrial integrity in conditions of FABP4 KD and FAO inhibition. Seventy to eighty percent of untreated control cells had a medium Δ*Ψ*_m_ (represented by R1), which was significantly decreased by FAO inhibition and/or FABP4 KD (Figures 6c and d (right panel)). This change was due to (1) an increase of low Δ*Ψ*_m_ cells (represented by R2), likely due to activation of apoptosis (Figure 6d, middle panel) and (2) an increase of high Δ*Ψ*_m_ cells (represented by upper R1) (Figure 6d, left panel). Indeed, when FABP4 was silenced, we observed a significant increase of high Δ*Ψ*_m_ cells (Figure 6d, left panel). Etomoxir partially rescued the high Δ*Ψ*_m_ in FABP4 silenced cells. The occurrence of high Δ*Ψ*_m_ cells in FABP4 silenced conditions fits the observed increase in mitochondrial ROS, which likely originate from increased FAO and mitochondrial respiration.

## Discussion

In this study, we investigated FABP4 in EC metabolism and function, and its potential as a therapeutic target in antiangiogenic therapies. Therapeutic delivery of siRNA led to reduced endothelial FABP4 expression and significant reduction of tumour growth. *In vitro*, FABP4 KD led to activation of lipolysis and a significant increase of FAO, causing increased Δ*Ψ*_m_ and mitochondrial ROS production.

Endothelial FABP4 expression in normal and tumour tissues of ovary and brain has been reported.^11, 26^ It has been shown previously only that adipocyte FABP4 expression is important for promoting ovarian carcinoma.^10^ We observed that endothelial FABP4 expression *in vivo* was enhanced in DLL4-overexpressing tumour xenografts, demonstrating the *in vivo* induction of FABP4 directly by NOTCH1 signalling. DLL4-overexpressing tumours have increased perfusion and are insensitive to BEV treatment, as described previously.^14^ Importantly, endothelial FABP4 expression was insensitive to VEGFA inhibition alone, but was reduced when inhibition of NOTCH1 and VEGFA signalling was combined. This may indicate that apart from direct NOTCH1-mediated induction of FABP4, other pathways are important for the maintenance of FABP4 expression *in vivo*. VEGFA and DLL4/NOTCH1 blockade by BEV and DBZ increases tumour hypoxia.^14^ Interestingly, mRNA expression of FABP4 was downregulated in hypoxia *in vitro* (data not shown). Thus increased hypoxia in combined BEV and DBZ-treated tumours *in vivo* may be contributing to reduced FABP4 expression. As FABP4 is a key gene downstream of DLL4/NOTCH1 and indirectly of VEGF, by targeting it we may overcome NOTCH1-mediated resistance in a less toxic way than by using γ-secretase inhibitors.^27^

FABP4 was more abundant in vessels of low-grade than high-grade ovarian carcinomas. Recent studies on the effect of BEV in ovarian cancer have shown improvement of progression-free survival (39 vs 34 months), but not overall survival.^28^ Even good prognosis groups showed 74% progression at a median time of 17 months from the start of treatment and a median survival of 27 months. In a subgroup analysis it was found that patients with low-grade carcinoma and less aggressive tumour features showed reduced benefit from BEV treatment compared with those in the more aggressive, high-grade group.^28^ Based on our observations, tumours of the low-grade subgroup are likely to have high endothelial FABP4 expression. This subgroup could potentially benefit from FABP4 inhibition.

In high-grade carcinomas, FABP4 was specifically expressed in stroma-rich regions, whereas vessels directly adjacent to tumour cells did not express FABP4. The tumour architecture is a determinant of resistance to antiangiogenic therapy.^29^ Tumours in which nests of tumour cells are surrounded by well-developed stromal structures containing the majority of the vessels are less responsive to single therapies targeting VEGFA, compared with tumours in which the vessels are embedded in the tumour mass. Thus, the stroma separates tumour cells from the vessels, potentially creating more mature vessels.^29^ NOTCH1, known for its limiting and stabilizing effect on angiogenesis, was co-expressed with FABP4 in these stromal regions, potentially promoting a more mature vasculature, and resistance to VEGFA-targeting therapies.

Previous work highlighted the role of FABP4 in adipocytes and adjacent ovarian cancer cells^11^ but a role of FABP4 in tumour vessels has not been studied directly. Nieman *et al.*,^11^ who studied ovarian tumour growth in FABP4−/− mice, attribute the decreased tumour burden solely to the lack of FABP4 expression in adipocytes and decreased FA metabolism in adjacent cancer cells. The researchers observed decreased microvessel density in tumours; the importance of endothelial FABP4 was, however, not investigated in that study.^11^ Our results show that siRNA-mediated FABP4 silencing in the vessels, while maintaining FABP4 in adipose tissue, leads to substantial loss of vessels and reduced tumour growth. This, in addition to effects of FABP4 on FA metabolism and mitochondrial ROS in ECs *in vitro*, strongly supports a role of FABP4 in tumour angiogenesis.

**Figure 7:**
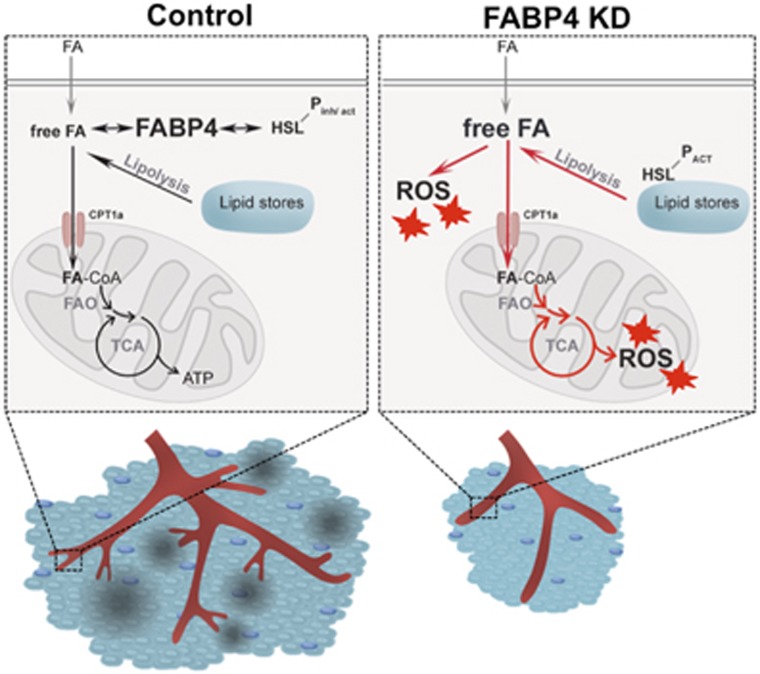
Proposed model for functional mechanism. When FABP4 is expressed in blood vessels (control), FABP4 binds to intracellular free/unbound fatty acids (FA), and provides feedback inhibition to hormone-sensitive lipase (which is phosphorylated at sites regulating both inhibition and activation of HSL, HSL-P_inh/act_) by controlling the balance between lipolysis, FA uptake and oxidation (entry of FA into mitochondria via carnitine-palmitoyl transferase 1a, CPT1a). When FABP4 is knocked down (FABP4 KD), free/unbound fatty acids in the cell are in excess. FABP4 loss leads to increased lipolysis, indicated by increased activating phosphorylation of HSL (HSL-P_ACT_), likely resulting in increased free FA availability. This leads to upregulation of rescue mechanisms such as the increase of the rate of FA oxidation (FAO) and potentially tricarboxylic acid (TCA) cycling and oxidative phosphorylation. Consequently, FABP4 loss is accompanied by increased mitochondrial and total ROS production and decreased cell viability, leading to reduced angiogenic activity and tumour growth, but no change in macrophage (dark blue) infiltration *in vivo*. The reduced tumour size is accompanied by less hypoxia and necrosis (in grey) in the tumour.

Adipocytes of FABP4−/− mice have decreased rates of lipolysis and accumulate intracellular free FAs.^8^ Endothelial FABP4 preserves the controlled metabolism of FAs in ECs, by keeping the intracellular release and degradation of FAs in balance (Figure 7). We show for the first time that HSL, a key lipolytic enzyme, is highly expressed in ECs. The expression of endothelial FABP4 was required for controlling HSL and thus lipolytic activity. Similarly, *PPARG* mRNA induction in FABP4 KD cells may have represented an attempt to (a) compensate for a lack of activating ligands or (b) channel excess free/unbound FAs into lipids.

FABP4 silenced cells have increased FAO. This may serve to remove excess free FAs resulting from increased lipolytic activity when FABP4 is lacking. We further hypothesize that activated FAO in FABP4 KD ECs is directly contributing to increased mitochondrial ROS production. The increased oxidation of FA leads to a higher Δ*Ψ*_m_, indicating enhanced electron transport chain activity, electron leakage and oxygen reduction. Since total ROS production was only partially inhibited by the superoxide dismutase mimetic MnTMPyP, FABP4 loss may generate cytoplasmic ROS also by increasing the levels of unbound FAs, leading to activation of ROS generators such as NADPH oxidases in the cytoplasm.^6, 30^ An increase of ROS production may reduce the ability of ECs to proliferate, sprout and form vascular networks.

Our data show that FABP4 is required for angiogenesis and an important target in tumour angiogenesis, especially in tumours that are low grade, rich in stroma and embedded in FA-rich tissues. RNA-based therapies that enable tissue-specific delivery are currently being tested in clinical trials.^31^ Our study provides a rationale for testing vascular endothelium-targeted delivery platforms with FABP4 siRNA to inhibit tumour growth.

## Materials and methods

### Cell culture

All cells were routinely tested for mycoplasma contamination. Primary human umbilical ECs were purchased from Lonza, Visp, Switzerland and cultured in endothelial growth medium 2 (Lonza). At least three different pools of 3–5 donors were used for experiments. SV40-transformed mouse ECs (SVEC4-10) were from ATCC (Manassas, VA, USA). SKOV3 cells used to obtain SKOV3 ip2 cells were from ATCC and cell line authentication was routinely performed.

### RNA interference

Reverse transfection of siRNA duplexes (20 nm) was performed using Lipofectamine RNAiMax (Invitrogen, Carlsbad, CA, USA) according to the manufacturer's instructions. Two different ON-TARGETplus siRNAs targeting *FABP4* (Dharmacon, Lafayette, CO, USA) were used (#1, targets 5′-AUACUGAGAUUUCCUUCAU-3′ #2, targets 5′-GGUGGAAUGCGUCAUGAAA-3′) and the ON-TARGETplus non-targeting pool (Dharmacon).

### Gene expression analysis

RNA extraction was performed using Trizol reagent (Invitrogen) according to the manufacturer's instructions. Reverse transcription (RT) was performed using the High Capacity cDNA RT kit (Applied Biosystems, Life Technologies, Bleiswijk, Netherlands). QrtPCR was performed using the SensiFAST SYBR No-ROX Kit (Bioline, London, UK). Raw data were analysed using the 2(−ΔΔct) method, using *ACTB* as a housekeeping gene. QrtPCR primer sequences (Invitrogen) were *hFABP4* (forward 5′-ACGAGAGGATGATAAACTGGTGG-3′, reverse 5′-GCGAACTTCAGTCCAGGTCAAC-3′); h*PPARG* (forward 5′-GGGGTGATGTGTTTGAACTTG-3′, reverse 5′-GACAGGAAAGACAACAGACAAATC-3′); m*FABP4* (forward 5′- GGATGGAAAGTCGACCACAA-3′, reverse 5′-TGGAAGTCACGCCTTTCATA-3′) and *ACTB* (forward 5′-GAGGAGGCACCGGTAAATG-3′, reverse 5′-GTCACTCACTGGGACATAGGC-3′).

### Protein expression analysis

Immunoblotting was performed using the Novex NuPAGE SDS-PAGE Gel and blotting system (Invitrogen). Antibodies were anti-total HSL, anti-p565 HSL and anti-660 HSL (sampler kit #8334; Cell Signalling Technology, NEB, Herts, UK), anti-FABP4 (HPA002188; Sigma-Aldrich, St Louis, MO, USA) and anti-β-actin-peroxidase (Sigma-Aldrich).

### FA accumulation and oxidation assay

Briefly, FA accumulation and oxidation were measured in HUVEC in endothelial growth medium 2 containing 50 μm carnitine and 500 μm bovine serum albumin-conjugated oleic acid (OA, both Sigma-Aldrich), and 0.5 μCi/ml [U-14C]-OA (Perkin Elmer, Boston, MA, USA). To measure FA accumulation, cells were lysed after 3 h of incubation. To measure FAO, 3 n KOH, in a separate container that was inserted into the tissue culture flask, was used to capture CO_2_. Sixty per cent perchloric acid (Sigma-Aldrich) was injected to capture intracellular CO_2_, as described previously.^10^ Etomoxir (50 μm; Sigma-Aldrich) was used to inhibit FAO.

### Lipid droplet quantification by FACS

Neutral and polar lipids in PFA-fixed cells were stained with the fluorescent dye LD540 (kindly supplied by Dr C Thiele, LIMES Life and Medical Sciences Institute, Bonn, Germany) and analysed by flow cytometry.

### Cell viability assay

Transfected cells were exposed to 10% Trypan blue solution (Life Technologies, Carlsbad, CA, USA). Total cell number and trypan blue positive cells were determined using the Cellometer Auto T4 cell counter and software according to the manufacturer's instructions (Nexcelom Bioscience LLC, Lawrence, MA, USA).

### Measurement of total and mitochondrial ROS

Total ROS levels were determined by 2′,7′-dichlorodihydrofluorescein diacetate (H2-DCFDA; Life Technologies); mitochondrial ROS levels were determined by MitoSOX (Life Technologies). ROS content was measured by flow cytometry. Manganese (III) tetrakis(1-methyl-4-pyridyl)porphyrin pentachloride (MnTMPyP, 12.5 μm) was from Santa Cruz Biotechnology (Dallas, TX, USA).

### Measurement of mitochondrial membrane potential (Δ*Ψ*_m_)

Δ*Ψ*_m_ was measured using the BD MitoScreen (JC-1) kit (BD Biosciences, Franklin Lakes, NJ, USA) according to the manufacturer's instructions. JC1 is a cationic dye that accumulates in the mitochondria, driven by Δ*Ψ*_m_. Briefly, cells were grown as indicated and incubated in 1.25 μm JC1 and analysed by flow cytometry. The gating strategy was based on the presence of three cell populations with high (upper R1) versus medium (R1) versus low (R2) emission in the PE channel.

### Scratch wound assay

HUVEC were grown to confluence on ImageLock 24-well plates (Essen BioScience, Ann Arbour, MI, USA). The scratch was introduced with a woundmaking tool (WoundMaker; Essen BioScience) and imaged with the Incucyte (Essen BioScience). The percentage of wound closure relative to the control (70–80% closure) was measured.

### Sprouting assay

HUVEC spheroids were grown in hanging drops containing 2.5 mg/ml methylcellulose (Sigma-Aldrich), embedded in Gibco Geltrex Basement Membrane Matrix (Life Technologies) and incubated for 48 h.

### Immunohistochemistry

Paraffin-embedded cell pellets or tissue sections were retrieved, dewaxed and rehydrated according to standard procedures. m/hFABP4 was stained with anti-FABP4 (HPA002188; Sigma-Aldrich) (Supplementary Figure S2), mFABP4 in FABP4-siRNA-treated xenografts was stained with anti-FABP4 (D25B3; Cell Signalling Technology). Others were rabbit Ki67 (M7240; Dako, Glostrup, Denmark), rabbit CA9 (M75; BD Biosciences) and goat NG2 (ab101808; Abcam, Cambridge, UK), CD31 and CD68 (BD Biosciences). Expression of CA9, Ki67 and NG2 was quantified on whole sections by using ImageJ software (US National Institutes of Health, Bethesda, MD, USA). Expression of CD31 and CD68 was quantified by using 15 random fields of slides at × 100 magnification.

### DLL4-overexpressing U87-xenograft selection and scoring

Formaldehyde fixed-paraffin embedded sections of full sets of EV and DLL4-overexpressing U87 xenografts treated with DBZ and/or BEV were obtained as described previously (*n*=5 per group).^14^ Vascular FABP4 was scored and a combined intensity and extent quick score was calculated.

### Serous ovarian tumour sample selection and scoring

Tissue microarrays of serous borderline and high-grade ovarian carcinomas and samples of paired primary and metastatic serous ovarian carcinoma were collected prospectively within a translational study that was ethically approved (Number 11/SC/014, Berkshire NRES Committee) and all subjects gave informed consent as appropriate. Vascular FABP4 in tissue microarrays was scored by Dr Ioannis Roxannis, Oxford University Hospitals, and a combined intensity and extent quick score was calculated. Vascular FABP4 in paired samples was scored using Aperio ImageScope and IHC Image analysis software (Leica Biosystems, Milton Keynes, UK), reporting the normalized strong positive score.

### Orthotopic *in vivo* model of ovarian cancer

Eight- to 12-week-old female athymic nude mice (NCr-nu) were purchased from Taconic farms (Hudson, NY, USA) and cared for in accordance with the guidelines set up by the American association for Accreditation of Laboratory Animal care and the U.S. Public health Service Policy on Human care and Use of Laboratory Animals. All *in vivo* experiments and protocols were approved by MD Anderson's Institutional Animal care and Use Committee. For SKOV3 ip2 cell injections, 1 × 10^6^ cells were injected directly into the ovary.

Control or mouse *FABP4* siRNAs (#1, target sequence 5′-CCGAGAUUUCCUUCAAACU-3′ #2, target sequence 5′-ACCAUCCGGUCAGAGAGUA-3′ supplementary #1, target sequence 5′-GGAAGGUGAAGAGCAUCAU-3′ supplementary #2, target sequence 5′-CUGGGCGUGGAAUUCGAUG-3′ from Sigma-Aldrich) were incorporated in chitosan nanoparticles as described previously.^19^ Briefly, chitosan was dissolved in 0.25% acetic acid. The nanoparticles were spontaneously formed after adding tripolyphosphate (0.25% w/v) and siRNA (1 μg/μl) under constant stirring at room temperature. The nanoparticles were incubated for 40 min at 4 °C, centrifuged at 12 000 r.p.m. for 40 min at 4 °C and stored at 4 °C until use.

Twice weekly injections of 3.5 μg/100 μl control siRNA-CH, sequence #1-CH and sequence #2-CH were started after 1 week (10 mice/group). The mice were observed daily for adverse effects and killed when any of the mice seemed moribund. Mouse weight, tumour weight and number of nodules were noted. Tissue specimens were fixed with formalin or were snap frozen.

### Statistical analysis

Results are expressed as mean±standard deviation (s.d.) or standard error of the mean (s.e.m.) as indicated. *In vitro* experimental data are shown as mean of at least three biological replicates. For animal experiments, 10 mice were assigned per treatment group. This sample size gave 80% power to detect 50% reduction in tumour weight with 95% confidence.^32^ After tumour inoculation, mice were randomly allocated into treatment groups. Mice that did not develop tumours were excluded from the analyses, unless otherwise mentioned. Investigators were blinded as to on which group they were performing necropsy. Statistical analysis was performed using one-way analysis of variance (ANOVA) and Holm–Sidak's multiple comparison test to compare groups of more than two, or two-sided Student's *t-*test to compare two groups (GraphPad Prism 6, La Jolla, CA, USA). It was assumed that the sampling distribution of the mean was normal. Variances were similar between groups that were statistically compared. *P<*0.05 was considered statistically significant.
