# Supplementary figures and images for: Antiangiogenic and tumour inhibitory effects of downregulating tumour endothelial FABP4
Source: Oncogene. 2016 Aug 29;36(7):912–21. doi: 10.1038/onc.2016.256 (PMC5318662; doi:10.1038/onc.2016.256)

Figure S1

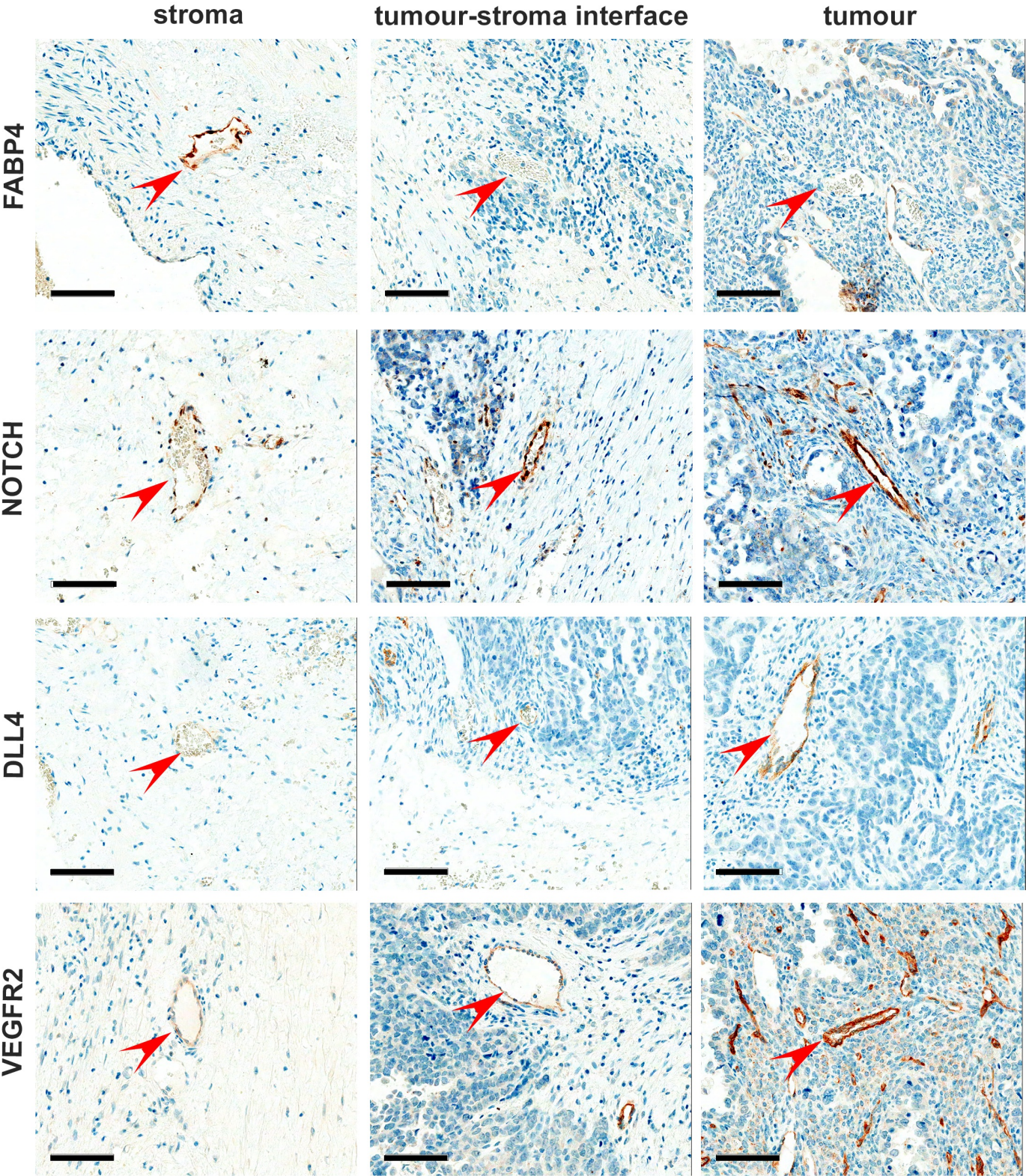

Supplement: Supplementary Figure 1 [file onc2016256x1.pdf]

**Figure S3**

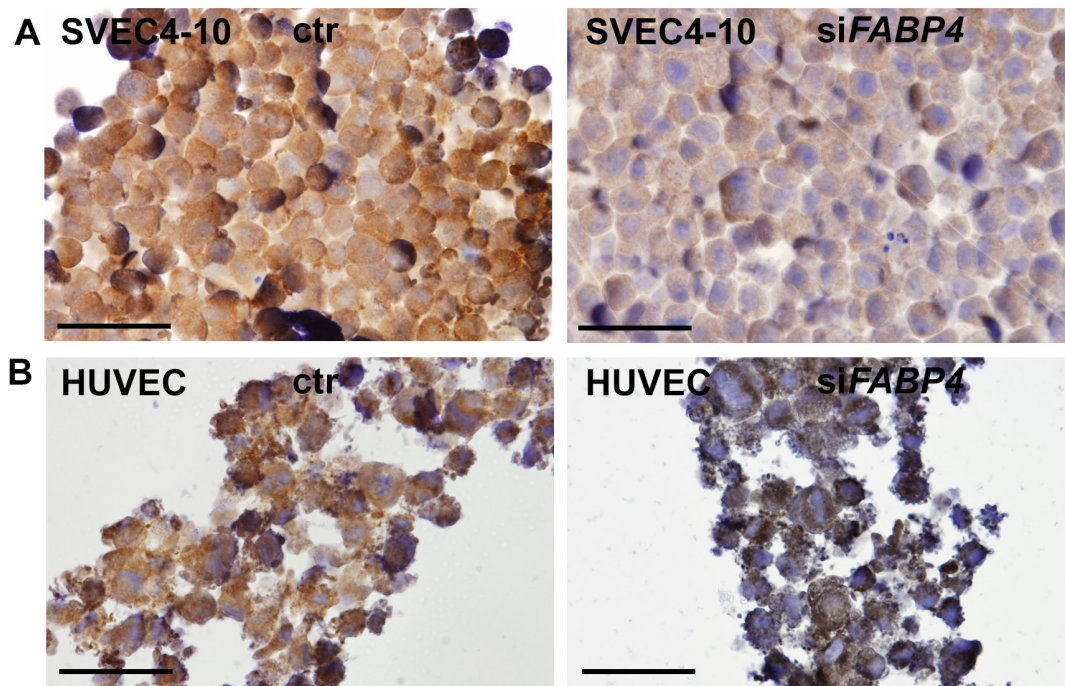

Supplement: Supplementary Figure 3 [file onc2016256x2.pdf]

Figure S3

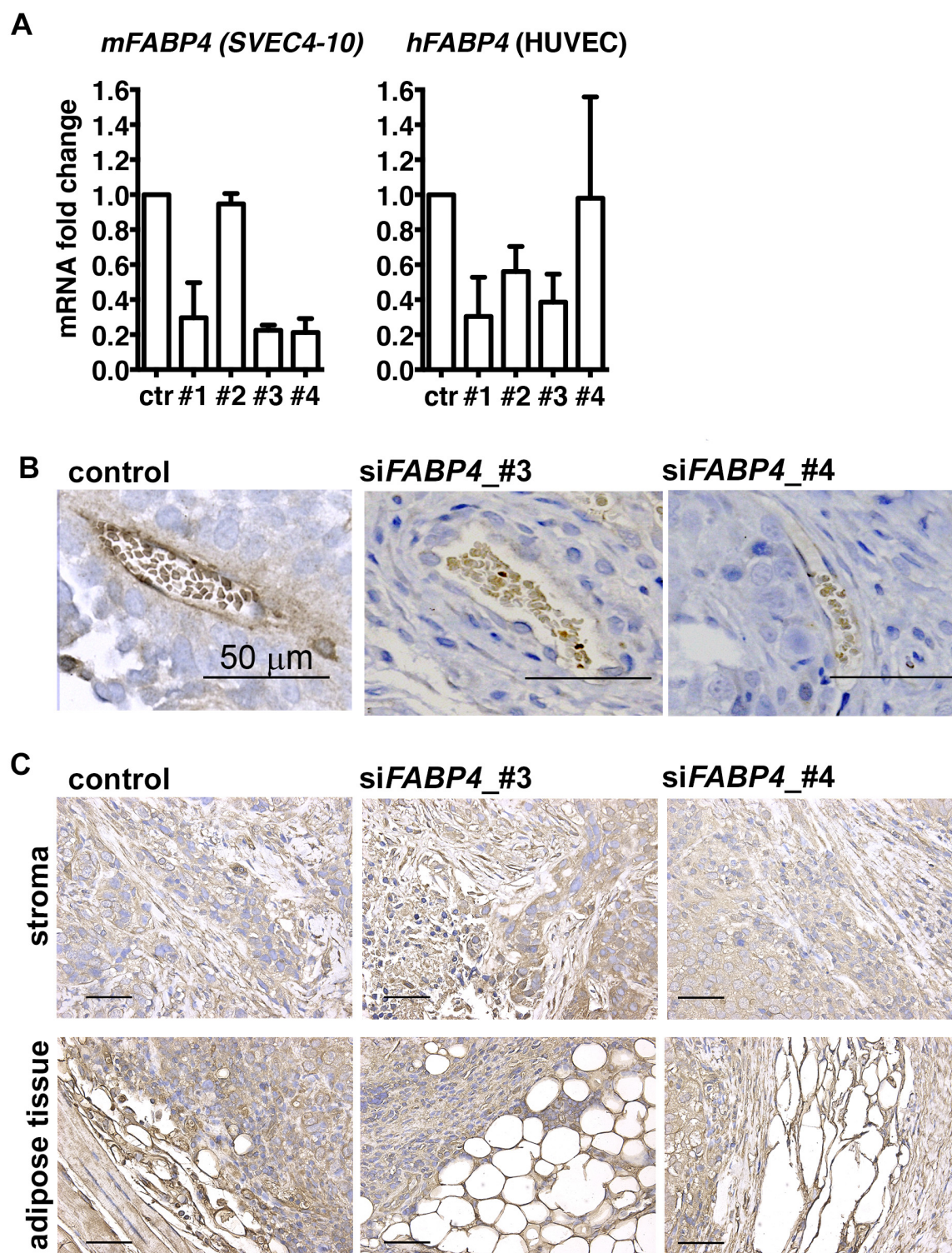

Supplement: Supplementary Figure 3 [file onc2016256x3.pdf]

Figure S4

A control

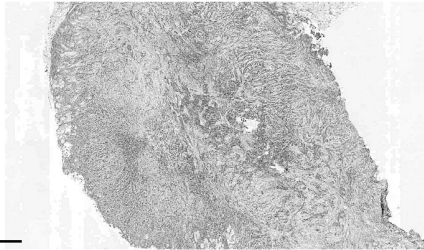

siFABP4-#1

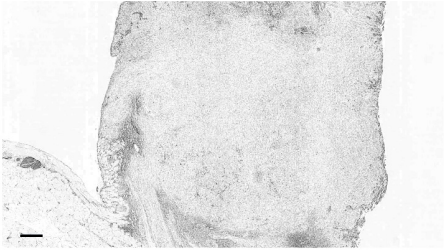

siFABP4-#2

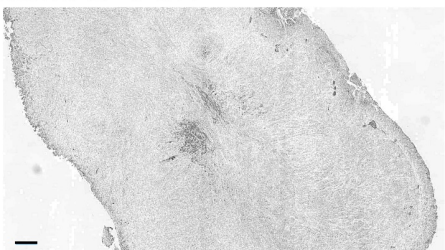

B

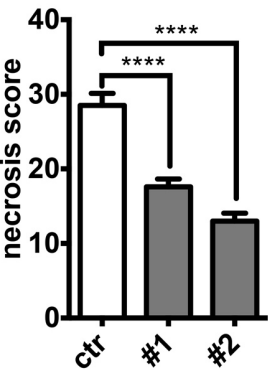

Supplement: Supplementary Figure 4 [file onc2016256x4.pdf]
